# A Customized Energy Management System for Distributed PV, Energy Storage Units, and Charging Stations on Kinmen Island of Taiwan

**DOI:** 10.3390/s23115286

**Published:** 2023-06-02

**Authors:** Hsi-Chieh Lee, Hua-Yueh Liu, Tsung-Chieh Lin, Chih-Ying Lee

**Affiliations:** 1 Department of Computer Science and Information Engineering, National Quemoy University, Kinmen County 892, Taiwan; 2 Department of Architecture, National Quemoy University, Kinmen County 892, Taiwan; 3 Department of Horticulture and Landscape Architecture, National Taiwan University, Taipei 106, Taiwan

**Keywords:** distributed PV, energy management system, energy storage units, charging piles, smart grid, redundancy, IoT, Home Assistant, low-carbon island, Kinmen

## Abstract

Kinmen, the famous Cold War island also known as Quemoy, is a typical island with isolated power grids. It considers the promotion of renewable energy and electric charging vehicles to be two essential strategies to achieve the goal of a low-carbon island and smart grid. With this motivation in mind, the main objective of this study is to design and deploy an energy management system for hundreds of current PV sites distributed on the island, energy storage systems, and charging stations on the island. In addition, the real-time acquisition of the data for power generation, power storage, and power consumption systems will be used for future demand and response analysis. Moreover, the accumulated dataset will also be utilized for the forecast or prediction of renewable energy generated by the PV systems or power consumed by the battery units or charging stations. The results of this study are promising since a practical, robust, and workable system and database are developed and implemented with a variety of Internet of Things (IoT), data transmission technologies, and the hybrid of on-premises and cloud servers. Users of the proposed system can remotely access the visualized data through the user-friendly web-based and Line bot interfaces seamlessly.

## 1. Introduction

### 1.1. Climate Responsibility and Energy Generation of Kinmen, Taiwan

**Figure 1:**
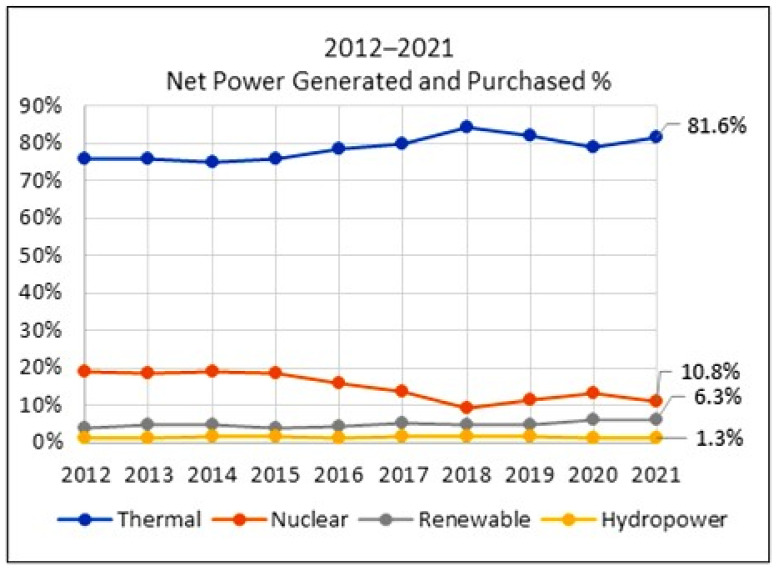
The net power generated and purchased by Taiwan’s Taipower Company, a state-owned exclusive enterprise.

From Taiwan’s perspective, three forces firmly push the renewable energy strategy forward. First, as a member of a global society, Taiwan provided its Intended Nationally Determined Contribution (INDC) on the 17 September 2015, including targets to achieve a 50% reduction below the BUA GHG emission level by 2030 [1]. Furthermore, Taiwan has demonstrated its commitment to achieving net zero by 2050 [2] through concrete actions, including implementing the Climate Change Response Act [3] in response to the 2021 26th Session of the Conference of the Parties (COP26), the U.N. climate conference held in Glasgow. Second, from an energy source viewpoint, Taiwan’s dependency on imported energy was 97.5% in 2020 [4] and even higher over the past 10 years. Looking into the composition of net power generated and purchased energy in 2021 [5], thermal energy dominated at 81.6%, as shown in Figure 1. It is apparently a risk regarding energy dependency and diversity. Third, Taiwan is still ambitious to strive for the vision of a nuclear-free homeland in 2025 with a clear energy target: 50% by natural gas, 30% by coal, and 20% by renewable energy. For the aforementioned goals, it is clear that promoting low-carbon renewable energy plays an essential role in achieving INDC and the nuclear-free vision and further balancing energy generation dependency and diversity.

Kinmen is an outlying island of Taiwan with a 150 km^2^ area, which has an isolated power grid for its electricity supply due to a distance of 248 km from western Taiwan. In Kinmen’s history, it had 43 years of being front-line against Communists until the abolishment of the military administration in 1992 [6]. With the gradually improved relationship between Taiwan and China and direct transportation across the national border, more and more tourists came to the Kinmen islands, resulting in higher and higher energy demand. Since 2013, Kinmen has been selected as a demonstrated low-carbon island by Taiwan Executive Yuan, and visions and strategies were set to reach zero carbon by 2030 [7]. The installation of renewable energy stations and low-carbon transportation have become two of the main strategies to achieve these goals.

In 43 years of military administration, this front-line constructed many distributed military facilities across the whole Kinmen islands, which were gradually released or abandoned since the troops left. From the analysis of [8,9,10,11], a distributed renewable energy power grid integrated with those facilities is a suggested and suitable strategy to fulfill Kinmen’s low-carbon vision.

Compared with traditional power networks, the smart grid is an advanced electricity platform that emphasizes two-way communication based on digital information technology. Key elements of an advanced smart grid include bulk electricity generation, demand response, distribution, utility companies, customers, transmission, service providers, and renewable energies [12]. Among these, sustainable analysis and management of data and information generated along with all activities is one of the most critical and valuable measures to get system efficiency and constant improvement.

In September 2021, the total installed PV capacity in Kinmen reached about 10.7 MW. While a distributed PV and energy storage system has become an essential approach for the Kinmen local government to move the low-carbon island vision forward, a reliable monitoring and data acquisition system that can constantly work for future data analysis of energy generation and efficiency under different circumstances is needed for this goal of a low-carbon island.

### 1.2. Remote Real-Time Monitoring and Controlling System for Distributed PV and Energy Storage Stations

Rahman et al. [13] conducted a very detailed review of different monitoring systems for PV since 1994, including RTAI, ZigBee, DAQ, SCXI, PIC, PLC, etc., in terms of their fundamental features, architecture, performance, and budget. Some of these remote systems further embrace the Internet of Things, web applications, and cloud platforms. The value of gathered data includes the sustainable status of the PV system, failure or error detection, and warning notification.

Al-Fuqaha et al. [14] reviewed the overview of the most relevant architectures and protocol standards for IoT. This study summarized the five-layer IoT model as the most functional architecture to develop an IoT system: Objects, Object abstraction, Service Management, the Application Layer, and the Business Layer. The Application layer relies on high computational machine resources. IoT functionality includes Identification, Sensing, Communication, Computation, Service, and Semantics. Challenges of Availability, Reliability, Mobility, Performance, Management, Scalability, Interoperability, and Security Privacy should be carefully considered in developing an IoT system.

In [15], a comparison of IoT sensor modules among Arduino, Raspberry Pi, PLC, and BeagleBone shows perspectives of data handling, cost and module size, and coding language. Ansari et al. concluded that Raspberry Pi is the most recommended due to its extension capability.

**Figure 2:**
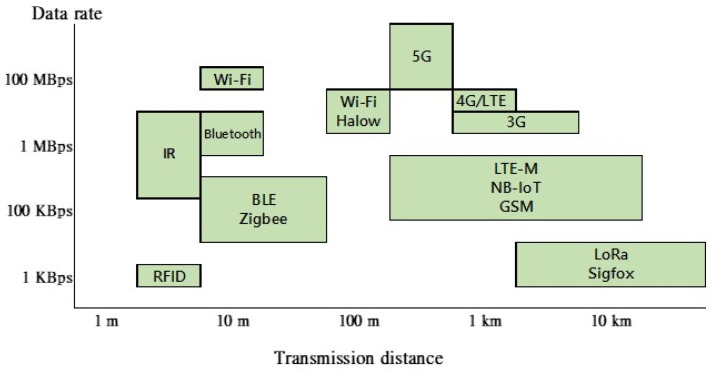
Comparison of different wireless technologies in terms of data rate and transmission distance.

Plenty of research has explored this field regarding local IoT networks and remote system connections. The popular wireless communication technologies used by IoT are shown in Figure 2.

Belghith et al. [16] designed a remote monitoring system that features star architecture of sensors, GSM communication, and a human–machine interface. Zego et al. [17] developed a wireless network to send sensed data to a local Raspberry Pi server via Zigbee. Li et al. [18] proposed a local ZigBee network and GSM connection for PV monitoring and fault diagnosis. It consists of data acquisition, data gateways, and a monitoring website based on the PHP Laravel framework. In Low-Power Wide-Area Network (LPWAN) applications, LTE-M, Sigfox, LoRa, and NB-IoT were developed. Among them, LoRa and NB-IoT are the most promising. LoRa is used in [19] for long-range and low power consumption requirements. In another implemented study [20], an Arduino-based data logger was designed to integrate 3G communication to serve stand-alone PV sites. Ascensión et al. described detailed designed data logger specifications corresponding to the IEC61724 standard. In [21], ZigBee was used as a local sensor network. After that, a 4G gateway was used to connect the local network to the internet for remote real-time monitoring. Melo et al. [22] proposed LoRa and Wi-Fi as local wireless networks. The structure comprises three key parts: data loggers, a local IoT system, and a Web application for monitoring.

Key research and comparisons are summarized in Table 1.

Redundancy refers to the backup of the system to prevent service disruption due to single-point failure. Namely, redundancy is the measure to achieve a robust and reliable service system. In order to ensure system redundancy, extra replicated servers are created with the same functions, applications, and other important service components. Failover means seamlessly and automatically switching to prepared backup servers while the primary system is down. The purpose of failover is to reduce the impact when a system failure happens. To the best of the authors’ knowledge, no previous studies have ever explored this mechanism in the renewable energy field or established reliable systems with this approach.

Moniruzzaman et al. [23] proposed a reliable web system supporting continuous service even if a system component fails. This high-availability system features computer cluster and loading balancing deployment via a three-tier architecture consisting of a Linux virtual server, virtualization, and shared storage.

Nguyen et al. [24] analyzed a hospital MIS system and suggested integrating different load balance and failover strategies to sustain hospital services under heavy system workloads. This edge/fog-based system design evaluated three load balance techniques: probability, random, and shortest queue-based approaches with or without failover function at different layers.

The main objective of this study is to develop and deploy a robust, reliable, workable, and suitable IoT-based PV monitoring system specific to Kinmen as a significant approach to achieving zero-carbon and smart-grid visions. This monitoring system is implemented in Kinmen with coverage of more than 40 sites, which is about half of the whole PV installed capacity in Kinmen. It is capable of collecting and archiving real-time data into on-premises and cloud database servers with IoT subsystem support that leverages Home Assistant, an open source of IoT Hub, to monitor the status and electricity usage of appliances, power generation of PV panels, and charging stations for electric motorcycles.

The main contributions and novelty of this study are as follows.

- A reliable and workable system: the relevant solar power generation facilities covered in this study started as early as 2015, including self-generation and self-use, Feed-In Tariff (FIT) wholesale sales, and grid connection. The total number of monitoring sites in Kinmen reached 50 by early 2023, proving that this system is a long-term effective practical information system and a crucial demonstration of island-level independent power grids.
- Leveraged technologies: from the data acquisition perspective, this study covers several technologies corresponding to different facility environments and data sources, including wired networks, wireless networks, TCP/IP, HTTP, crawler, IoT technology, and cloud technology. It demonstrates that IoT and cloud technology can significantly facilitate and manage large-scale renewable energy facilities.
- Established dataset: data established and collected by this research are an essential dataset for future power generation and consumption research in the Kinmen area.
- Integration of distributed PV, energy storage, and charging stations: this research includes integrating electric vehicle charging stations, solar power generation, and energy storage, which is vital as leading pre-research on demand response and smart grid research in the future.
- Redundancy and failover design with a hybrid of on-premises and cloud systems: no previous studies have ever explored this field or established reliable systems with this approach.

## 2. System and Methods

This study designed and deployed a set of information systems for data acquisition and monitoring, which was applied to many distributed energy storage and renewable energy sites on a medium-sized island with an independent power grid as a basis for system security, performance, maintenance, and data technology development.

### 2.1. System Overview and General Description

**Figure 3:**
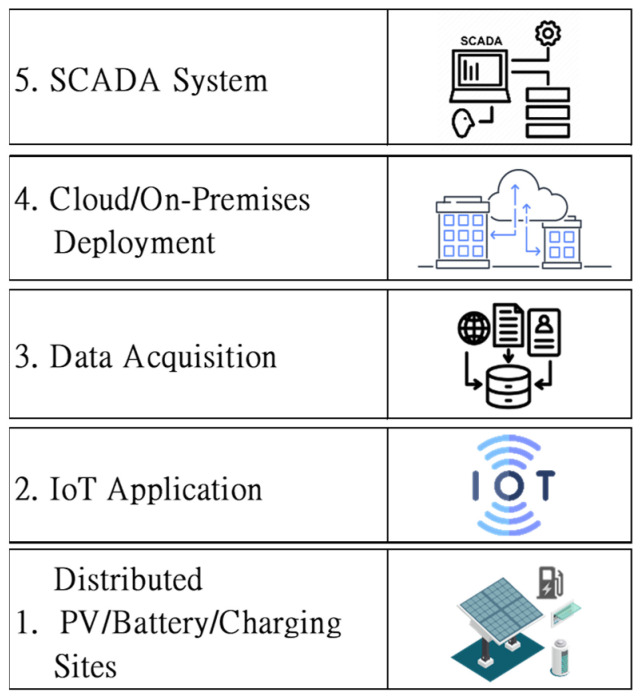
The high-level and five-layer architecture of the proposed system.

The overview of the proposed system, which aims to contribute a smart grid in Kinmen, is composed of five layers of critical functions, as illustrated in Figure 3. The first layer is distributed facility sites, including PV, battery, and charging stations, and it is the core of the green energy facilities of the whole project. The second layer is IoT, the front tier of the proposed monitoring information system, which is deployed to sense real-time data of the daily running facilities. The third layer is data acquisition, which is designed to get all real-time data back to on-premises servers. The fourth layer is the hybrid of cloud and on-premises deployment, which is capable of handling ample data information flow and designed with the perspective of redundancy and failover. The last layer is a custom SCADA system designed by this project with various friendly user interfaces.

**Figure 4:**
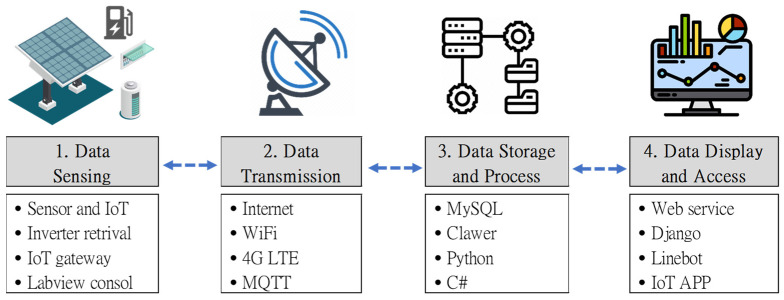
The four-layer architecture of the data processing flow of the proposed system.

**Figure 5:**
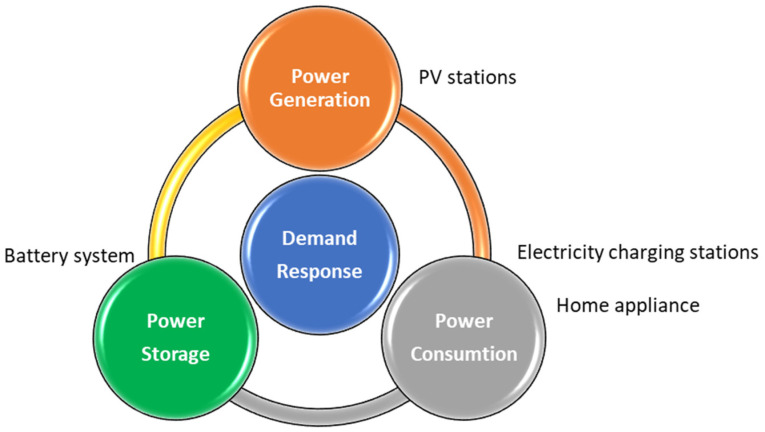
Purpose of dataset collection.

The most valuable element of this proposed system is the data. These facilities operate daily and generate real-time big data, which could be further analyzed and transformed into periodical reports, or as critical datasets for predictions in future uses. The four-layer architecture of data processing is explained in Figure 4, namely, data sensing, data transmission, data storage and process, and data display and access. For the long-term study of the following plan, demand response analysis, this plan is deployed mainly to collect three types of data, power generation, power consumption, and power storage, depicted in Figure 5.

**Figure 6:**
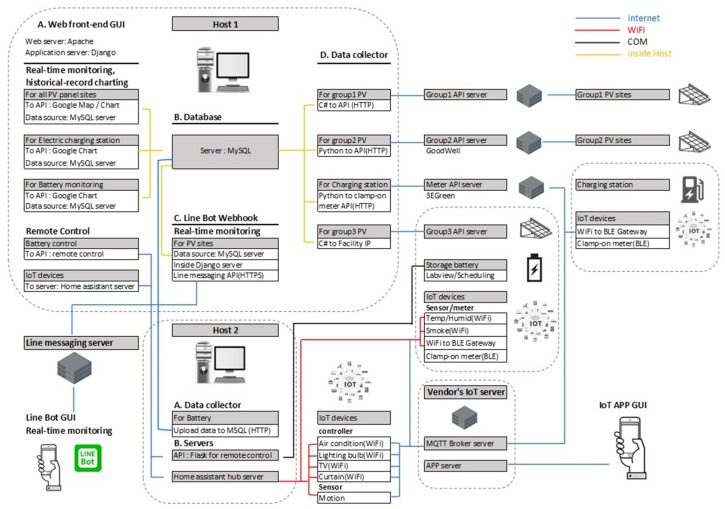
Low-level block diagram of on-premises deployment of the proposed system.

In addition to the overview mentioned above, Figure 6 presents low-level intra-system interactions, dataflow, network, interface, and user GUI, which is depicted based on on-premises deployment and will be explained in the following sections.

**Figure 7:**
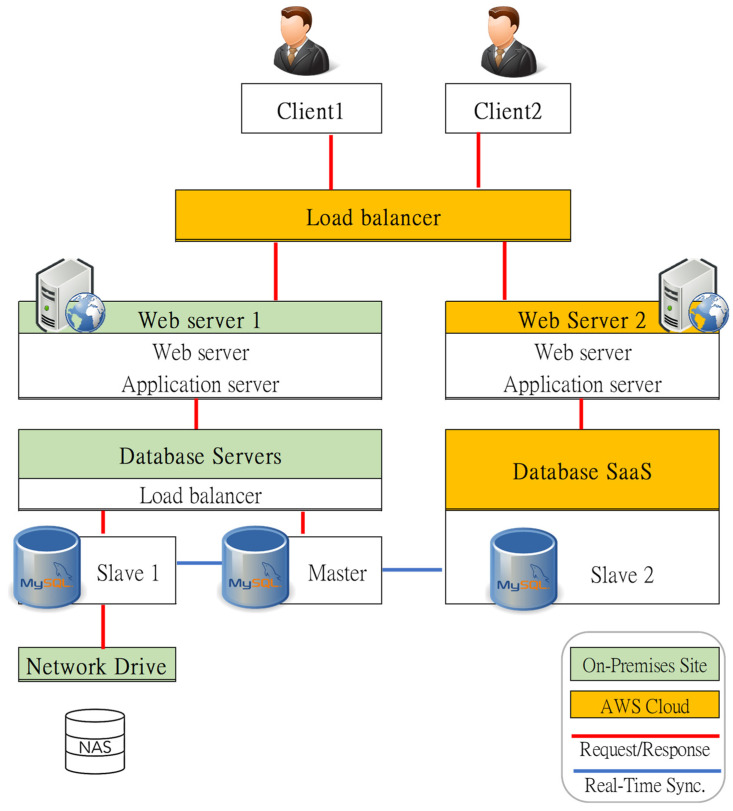
Cluster redundancy design based on a hybrid of cloud and on-premises deployment.

Redundancy and failover design are basic requirements for a sustainable and robust system. AWS cloud service is leveraged in the redundancy plan in this study. Figure 7 depicts the cooperation and backup among servers belonging to on-premises or cloud.

**Figure 8:**
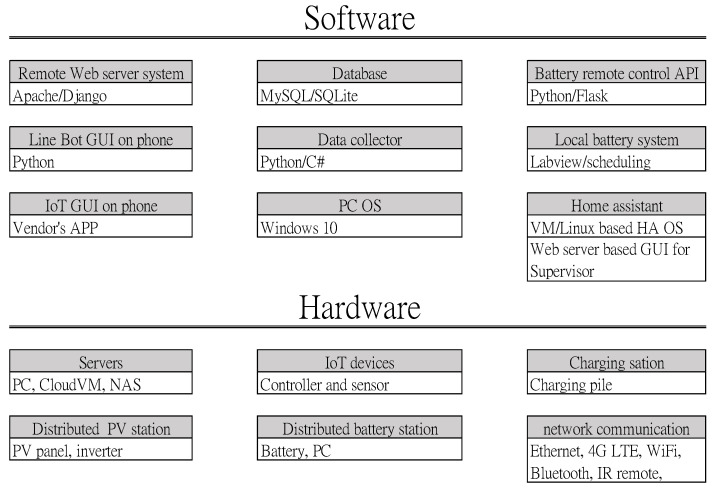
Key technologies of the software and hardware used.

Figure 8 shows key software and hardware technologies that serve the system per the site’s conditions and connection flexibility. Open-source software is leveraged as much as possible for better coding extensionality while hardware and facility are developed and deployed.

### 2.2. Detailed Design

As described in Figure 3 and Figure 4, the system operates within five-layer system architecture in which each subsystem interacts and four-layer data processing architecture in which data are generated, transmitted, stored, processed, and displayed. This section explains low-level activities and critical designs.

#### 2.2.1. On-Premises Remote Central Monitoring and Archiving Database System

- Web server

The SCADA, a custom web application as the monitoring and controlling core, can be remotely accessed from anywhere and at any time. It is designed with Python-based Django architecture and mainly leveraged with Google Maps and Google Chart APIs for site localization and statistics visualization. Regarding remote controlling, Python-based APIs were developed for front-end requests through HTTP. This server lives in Windows OS with an Apache web server in the production phase.

- MySQL master server

**Figure 9:**
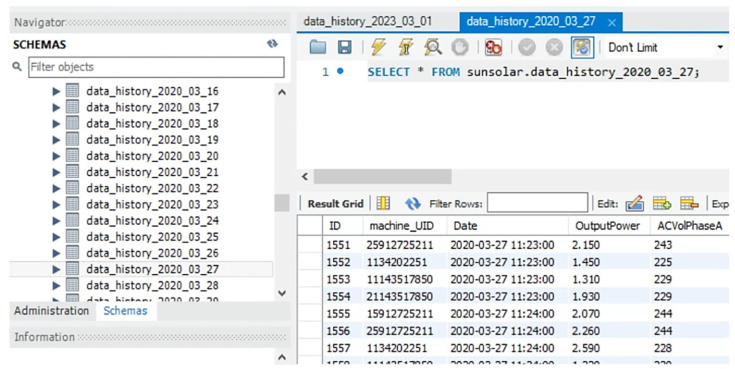
MySQL Workbench.

The MySQL server supports the back-end data archive and retrieval. The master is installed with the web server in one host for better transmission speed. The database application GUI example is shown in Figure 9. The earliest data were established in 2015.

- Line bot GUI

The Apache server uses an SSL certificate for the HTTPS channel. Line bot web-hook lives in Django with HTTPS support. Users can actively query from a smart device or passively receive daily reports via this automatic bot publication functionality.

- Data Collector

In Figure 6, group 1 PV is mainly state-owned facilities. A C# API was designed as a data collector for retrieving the data servers of this group. Meanwhile, a C# TCP/IP application was designed for direct connection to group 3’s PV inverters, which were designed without middle data servers. As for group 2’s PV and charging station, the crawler is used to collect from a third-party’s API from middle servers. These groups were built for different purposes at different times, so different data acquisition approaches are used to retrieve and observe their real-time data. Nevertheless, all data are finally archived in the same database with the same data format.

#### 2.2.2. IoT Hub, Local Database, and IoT Network

- IoT Hub

Home Assistant (HA), a popular hub tool for most IoT devices, was introduced as the IoT Hub, a Python-based open-source platform specific to smart-home applications. Dataflow between HA and IoT devices could be direct and local via LAN or indirect via external third-party API. The former is preferred because of privacy considerations. For the GUI of HA, users can access it via a web browser or smart device APP. In a LAN case, it may need VLAN to get a HA link to a different subnet, while VPN is required in order to be through the internet. This GUI is mainly for developers or system administrator access, not for regular users.

- Local Database

SQLite is used locally to work with HA. It also works as a data logger for IoT devices and local backup for the central MySQL database in case the internet is out of the connection.

- IoT Network

The wireless IoT controllers and sensors are connected to LAN via Wi-Fi, BLE, IR, or sub-1G. In case the facility site condition is complex for mentioned wireless or wired internet access, 4G LTE is used for internet connection, such as for stations in rural areas.

#### 2.2.3. PV, Battery, and Charging Stations

- PV station

**Figure 10:**
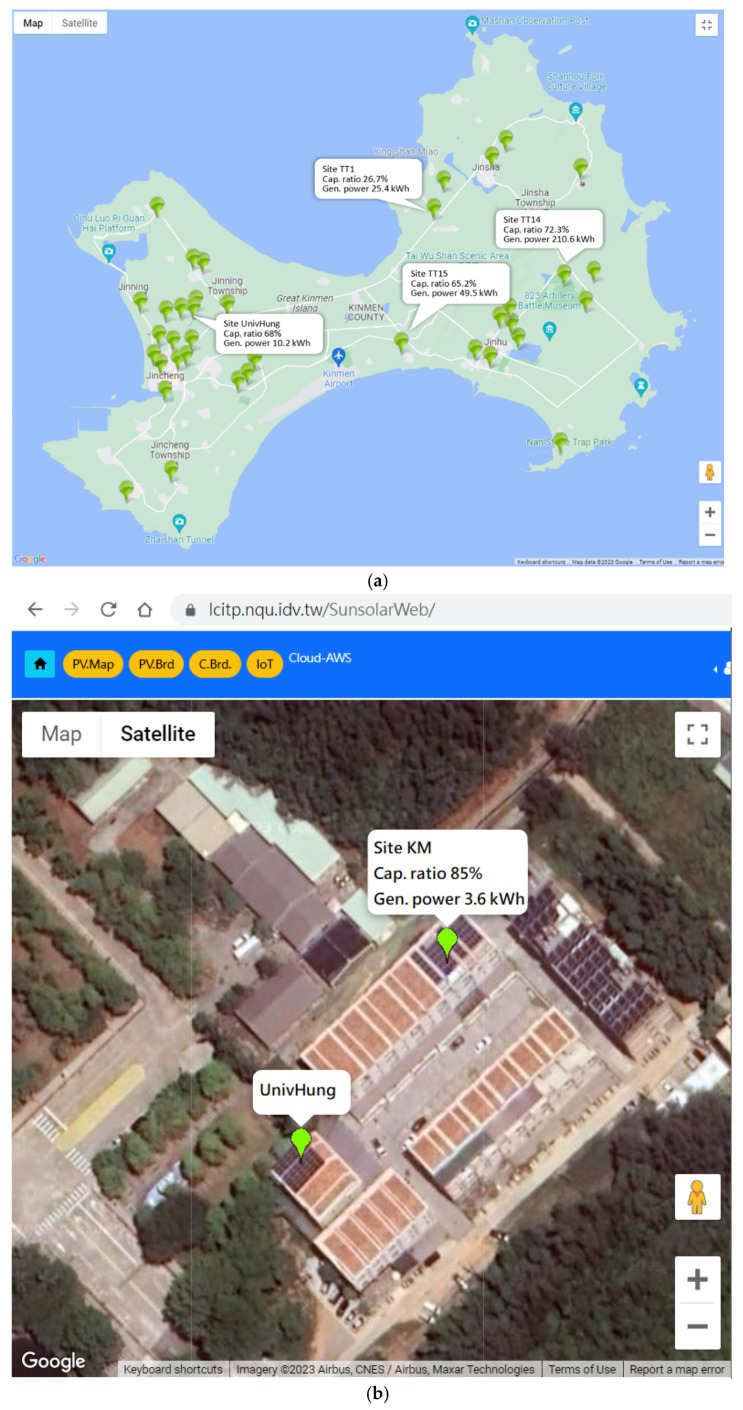
(**a**) All monitored PV sites in Kinmen are shown on Google Maps; (**b**) a clear PV-panel image on the roof of the monitored site.

The ongoing project continues to increase data collection of newly built PV stations in Kinmen. So far, the relevant data includes information from state-owned stations, privately owned resident stations, Taipower project stations, and Lab stations, among others. Some sites are based on FIT contracts, and some are for private use or research. The earliest sites have been running since 2015. The total installed and monitored capacity in this system is about 5 MW. More than 5 years of data from state-owned sites are incorporated. Figure 10a shows all monitored sites in the system via Google Maps, and Figure 10b shows one case with clear PV panels on the roof in satellite picture mode.

- Battery station

Distributed battery stations were added to this project in 2021, mainly for demand response research. Until now, one site stably runs for over a year with a 10 kWh storage capacity. The key components are a Windows PC, inverters, meters, and batteries inside this facility.

- Charging stations

**Figure 11:**
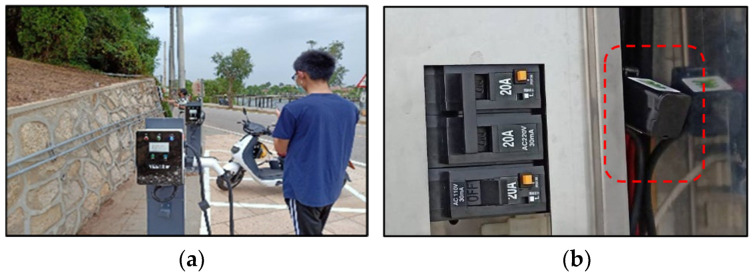
(**a**) Field picture with charging piles, (**b**) power distribution box with an IoT clamp-meter marked in red.

Kinmen has 65 state-owned free charging sites for electric motorcycles. It started monitoring the charging data from some newly built charging piles for vehicles and motorcycles in 2021. Figure 11 shows one newly built site in Kinmen National Park.

#### 2.2.4. Redundancy and Failover

- Cloud redundancy

In Figure 6, a cloud AWS VM is used for the replicated web server and load balancers. As to the Mysql database, SaaS database service is used as well. A load balancer contributes workload balancing and automatic web service failover functions.

- On-premises redundancy

The load balancer is used for database failover with a shared NAS drive. All Mysql servers get real-time synchronization by setting one master and two slaves.

#### 2.2.5. Software and Language

- Python 3.9

Python and open-source Python-based applications were mainly used for better integration, extensions, and sustainability, such as Django, Flask, HA, HTTP API, Line bot, data collector, and battery charging scheduling application.

- C# 10

**Figure 12:**
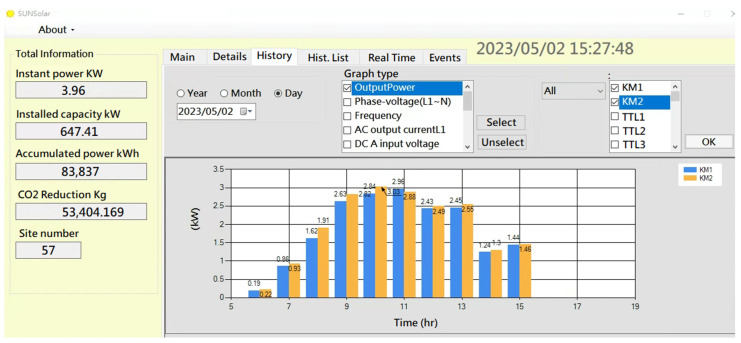
C# GUI for PV stations.

For the site group 1,2, a C# application was developed to work as the data collector. This application GUI is shown in Figure 12.

- Vendors’ APP for IoT device

This is a backup alternative to web GUI and HA GUI of deployed IoT devices. However, the disadvantage is privacy concerns due to data uploaded to third-party servers.

- Labview for battery module

The battery control console was designed by Labview.

- HA

A VM of Linux-based HA OS is used inside the Windows server. GUI for HA is used via web service. It is easy to access from anywhere with the internet.

#### 2.2.6. Hardware

- PC

Regular PCs are used with Windows OS for servers.

- IoT devices and network devices

Devices including smoke sensors, temperature and humidity sensors, motion sensors, clamp-on meters for electricity measurement, switches, curtain controllers, air conditioning controllers, and smart lighting bulbs were installed, as well as network devices including routers, Wi-Fi AP, Wi-Fi/BLE gateways, and infrared (IR) remote controllers.

- Facility Stations

The facility station mainly includes PV panels, inverters, batteries, and charging piles.

## 3. Results

### 3.1. PV Stations

**Figure 13:**
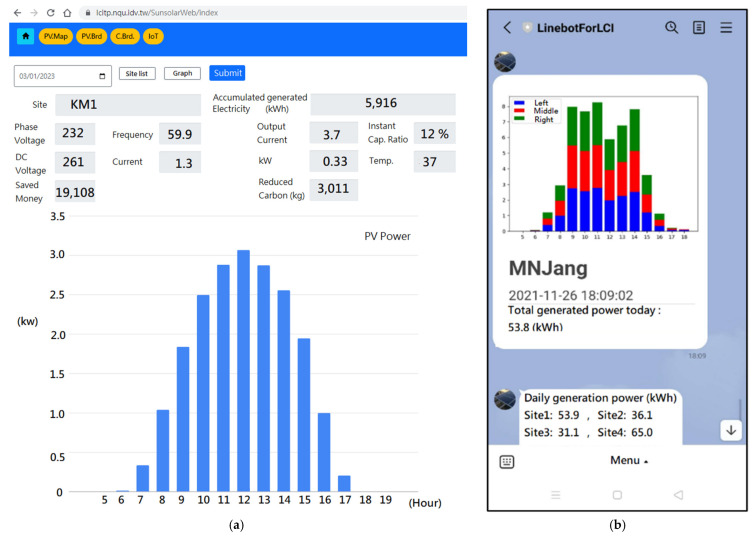
(**a**) Desktop web GUI, (**b**) smartphone line bot GUI. Both show PV daily data.

For PV site real-time monitoring and historical data review, users can accomplish this via web GUI on a desktop or line bot on smart devices, as shown in Figure 13. Users can actively or passively receive detailed daily data from the Line bot application (Figure 13b).

### 3.2. IoT Devices

**Figure 14:**
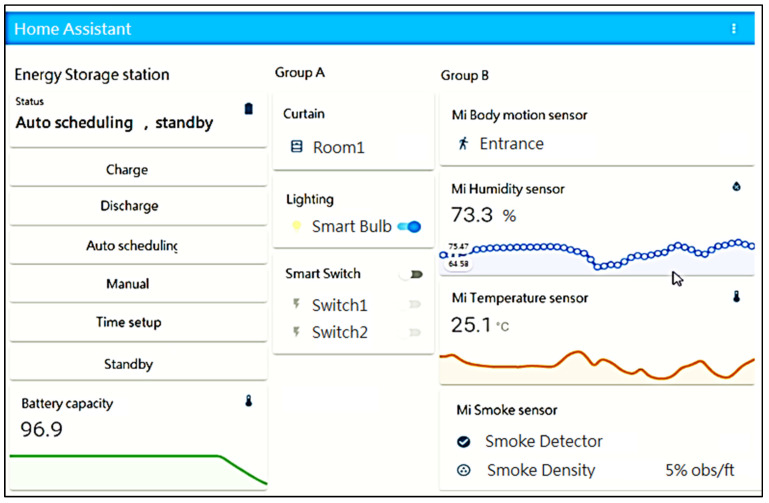
HA browser GUI for IoT device controlling and monitoring.

**Figure 15:**
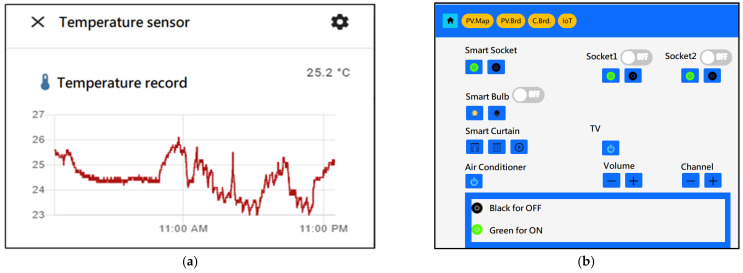
(**a**) HA temperature daily charting of IoT sensor, (**b**) Web browser GUI for IoT device control.

For IoT hub monitoring and controlling, users can accomplish this via HA GUI or web GUI as in Figure 14 and Figure 15. Due to the higher risk of battery operation, a temperature/humidity sensor and a smoke sensor were put inside the battery cabinet to monitor environmental security.

### 3.3. Battery Station

**Figure 16:**
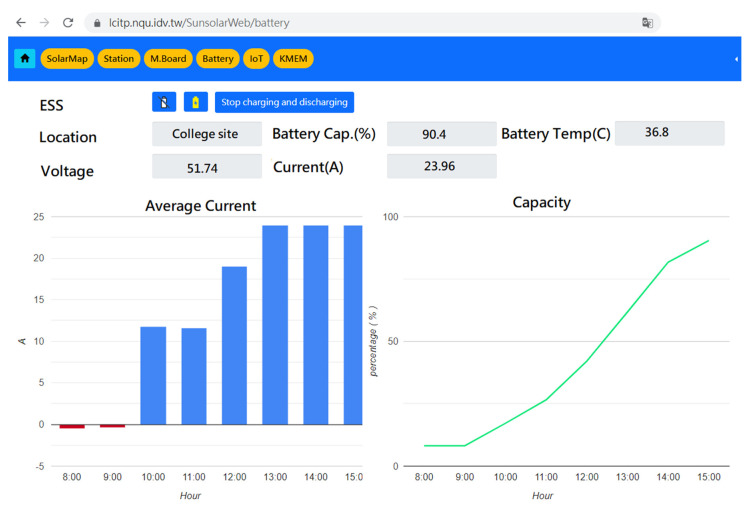
Web GUI for battery.

Battery monitoring and schedule control can be accomplished via HA GUI or web GUI as in Figure 16.

### 3.4. Redundancy and Failover Based on a Hybrid of On-Premises and Cloud Servers

The on-premises central hosts are located in a lab of Quemoy University. Typically, several power failures or internet disconnections happen each year. In the initial stage of the project, these events would lead to the web service going down or discontinuity of collected real-time data in database servers. Since the introduction of redundancy and failover mechanisms, the supporting servers are globally deployed on AWS with much less chance of being down in the meantime.

## 4. Discussion

SCADA mainly uses the Django web application and Home Assistant to monitor and control the facility and IoT devices. They are browser-based, so users can easily access them anywhere on any computer or smart device. It also provides Line Bot, which has “reply message” and “push message” functions as monitoring alternatives. The front-end service servers are deployed both on-premises and in the cloud as a redundancy design, and back-end database servers are deployed similarly. From the perspective of service accessibility, reliability, flexibility, and availability, this proposed system is much more comprehensive and functional than the cited research.

Facility stations in this study spread across the whole main island of Kinmen. Some of the stations downtown can be connected to the internet via wired or wireless methods, but some rural areas must use 4G LTE for wireless internet access. 4G LTE has higher quality and transmission rate than the other technologies shown in Figure 2 in case more extensive data transmission is needed, such as video surveillance.

Via scheduling setup, the energy storage system is beneficial to balance PV power generating fluctuation due to sunlight intensity and time-of-use rate mechanism. Electrical transportation is a sure trend for low-carbon policy. A good understanding of vehicle user charging behavior could contribute to stabilizing the power grid. None of the cited research has worked on integrating power generation, power storage, and power consumption.

The coverage of this work is more versatile than the cited research. Moreover, all collected data, system facilities, and approaches are beneficial for future demand response plans based on distributed virtual power plants. Good utilization of accumulated raw big data would make this system valuable and in line with the future smart grid vision.

Current limitations and future work:

- This study selected available methods for the IoT transmission approach but only explored some relevant technologies. It is believed there is room for optimization.
- Due to budget limitations and the availability of data sources, the quantity of energy storage facilities and wind energy generation stations is insufficient, and the monitoring data for power consumption is insufficient to produce an informative dataset for demand response analysis.
- Data value is based on good extraction and transformation. Although the current system can collect raw data and visualize it well, it needs to upgrade system capability further and integrate artificial intelligence models to make meaningfully data-driven predictions and optimize future demand response design to the automatic level of the machine-to-machine (M2M) by machine learning.

## 5. Conclusions

Kinmen is a resource-limited island with good solar and wind energy potential. The low-carbon trend is a must-do item to fulfill responsibility as a world member. Smart grid and low-carbon requirements could simultaneously move forward well with the support of a well-designed information system. Technically, only a system that can dynamically adjust demand response balance could make a smart grid possible.

In this work, a comprehensive monitoring and data collection system is well developed and deployed with versatile technologies corresponding to different environments and service requirements. With the redundancy deployment on a hybrid of on-premises and cloud systems, this robust, reliable, workable, and suitable IoT-based PV monitoring system specific to Kinmen is a practical approach to achieving zero-carbon and smart grid visions. Users can remotely access visualized data through the developed user-friendly web browser and Line bot. This implemented system collected and archived real-time data in terms of power generation, power storage, and power consumption since 2015 with IoT subsystem support to monitor site status and electricity usage of each site. The established dataset is essential for future power generation and consumption research in the Kinmen area.

The proposed system is on the way to integrating dataflow of distributed energy generation and storage, charging stations, and home electricity usage via IoT to make Kinmen a benchmark city with the smart grid.

## Figures and Tables

**Table 1:** Comparisons with cited research.

Title	Published Year	Data Log System	Monitoring System or SCADA	Data Transmission (LAN)	Data Transmission (to Internet)
Remote GSM module monitoring and photovoltaic system control [16]	2014	PIC18F4550 MCU	LabVIEW	Wired	GSM
A low-cost solar generation monitoring system suitable for Internet of Things [17]	2017	Raspberry Pi	Web APP	ZigBee	NA
Online monitoring system of PV array based on Internet of Things technology [18]	2017	DSP-TMS320F28335/ Raspberry Pi	Web APP	ZigBee/ Wi-Fi	Wired
An Alternative Internet-of-Things Solution Based on LoRa for PV Power Plants [19]	2019	Arduino/ Raspberry Pi	NA	LoRa	Wired
IoT Application for Real-Time Monitoring of Solar Home Systems Based on ArduinoTM With 3G Connectivity [20]	2019	Arduino UNO	ThingSpeak	Wired	3G
A Real-time Monitoring System Based on ZigBee and 4G Communications for Photovoltaic Generation [21]	2020	Cloud server	Web APP	ZigBee	4G
A Low-Cost IoT System for Real-Time Monitoring of Climatic Variables and Photovoltaic Generation for Smart Grid Application [22]	2021	Heltec Wi-Fi LoRa 32	Web APP	LoRa/Wi-Fi	Wired
Proposed system	--	Central/Cloud servers	Web APP	Wired/Wi-Fi/IR/BLE	Wired/ 4G LTE

## Data Availability

The data presented in this study are available on request from the corresponding author.

## Abbreviations

The following abbreviations are used in this paper:

AP	Access Point
API	Application Programming Interface
APP	Application
AWS	Amazon Web Services
BLE	Bluetooth Low Energy
DAQ	Data Acquisition
FIT	Feed-In Tariff
GUI	Graphical User Interface
HA	Home Assistance
HTTP	Hyper Text Transfer Protocol
HTTPS	Hyper Text Transfer Protocol Secure
IoT	Internet of Things
LoRa	Long Range
LPWAN	Low-Power Wide-Area Network
LTE	Long-Term Evolution
MCU	Microcontroller Unit
MQTT	Message Queuing Telemetry Transport
NAS	Network Attached Storage
TCP/IP	Transmission Control Protocol/Internet Protocol
PIC	Peripheral Interface Controller
PLC	Programmable Logic Controller
PV	Photovoltaic
RTAI	Real-Time Application Interface
SCADA	Supervisory Control and Data Acquisition
SQL	Standard Query Language
SaaS	Software as a Service
Wi-Fi	Wireless Fidelity
